# Supplementary material for: Earbox, an open tool for high-throughput measurement of the spatial organization of maize ears and inference of novel traits
Source: Plant Methods. 2022 Jul 28;18:96. doi: 10.1186/s13007-022-00925-8 (PMC9331584; doi:10.1186/s13007-022-00925-8)
Supplement: Supplementary file 5 — Additional file 5 Fig. S5. Illustration of the image correction applied to project the distances and positions of the reference points onto a hypothetical circular section of the ear. Orange circle, boundaries of the ear. The reference image measurement (dref) is corrected using the horizontal distances measured between its extreme reference points (black dots) and the center of the ear (dmax and dmin). The final corrected measured is an estimate of the length of the arc resulting from the projection of dref onto a hypothetical perfect circle of radius (Rear). [file 13007_2022_925_MOESM5_ESM.pdf]

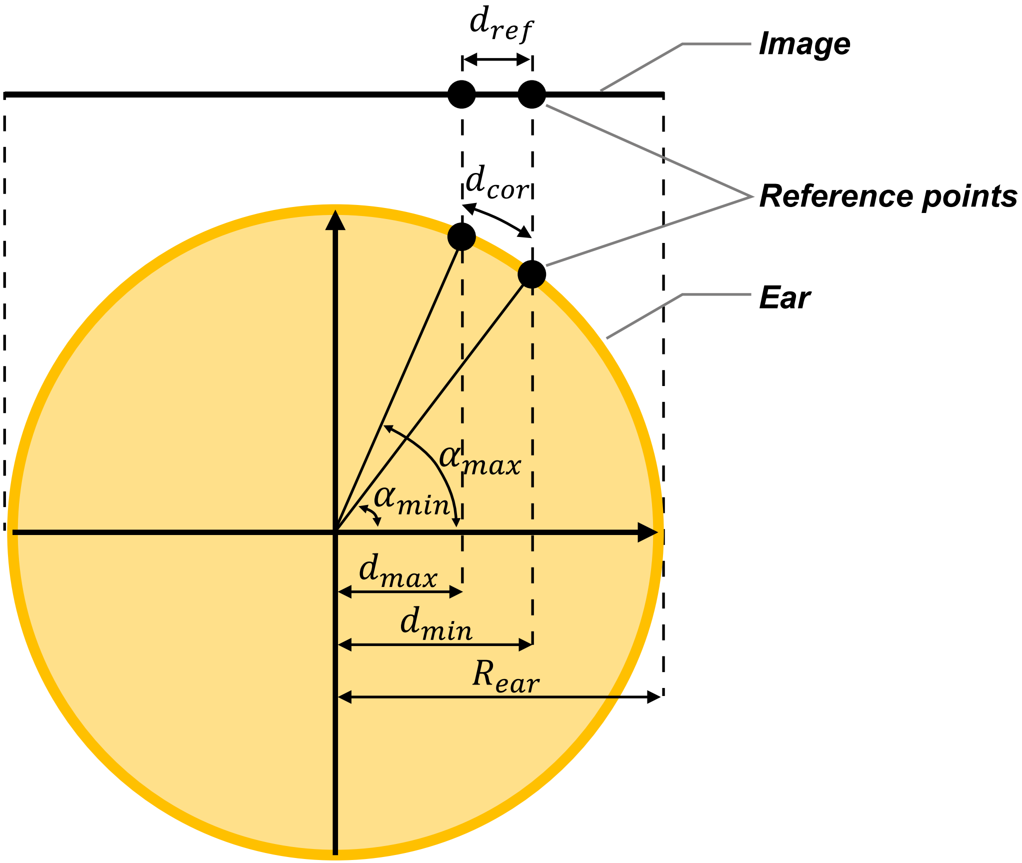

Corrected measurement: $d_{cor} = (\alpha_{max} - \alpha_{min}) * R_{ear}$

With: $R_{ear} = ear\ radius$, $\alpha_{max} = \cos^{-1}\left(\frac{d_{max}}{R_{ear}}\right)$ and $\alpha_{min} = \cos^{-1}\left(\frac{d_{min}}{R_{ear}}\right)$
