# Supplementary material for: Earbox, an open tool for high-throughput measurement of the spatial organization of maize ears and inference of novel traits
Source: Plant Methods. 2022 Jul 28;18:96. doi: 10.1186/s13007-022-00925-8 (PMC9331584; doi:10.1186/s13007-022-00925-8)
Supplement: Supplementary file 10 — Additional file 10 Table S1. Results of ANOVA testing the effect of Clusters of hydric conditions on mean ear length. The table was calculated using SPSS Software’s function ‘1-factor ANOVA’. Columns contain the values for Sum of squares, degrees of freedom (DF), Observed Fischer coefficient (Fobs) and the calculated significance, for between Groups (clusters) and inside groups (clusters) tests. [file 13007_2022_925_MOESM10_ESM.pdf]

	Sum of squares	DF	Fobs	Significance
Between Groups	2651,889	4	78,6	<,001
Inside Groups	5566,934	660		
Total	8218.802	664		
